# Characterizations of H4 avian influenza viruses isolated from ducks in live poultry markets and farm in Shanghai

**DOI:** 10.1038/srep37843

**Published:** 2016-11-29

**Authors:** Ying Shi, Hongrui Cui, Junheng Wang, Qiuyan Chi, Xuesong Li, Qiaoyang Teng, Hongjun Chen, Jianmei Yang, Qinfang Liu, Zejun Li

**Affiliations:** 1 Shanghai Veterinary Research Institute, Chinese Academy of Agricultural Sciences, Shanghai, People’s Republic of China

## Abstract

H4 avian influenza virus is one of the most prevalent influenza virus subtypes in birds. The evolution and pathogenicity of H4 AIV in domestic birds of China remain largely unclear. In the present study, a total of eight H4 AIV strains isolated in duck farm and live poultry markets (LPM) were characterized. Phylogenetic analysis indicated that these strains are divided into two groups in the Eurasian lineage. Eight genes of MH-2/H4N6 isolated from a duck farm were closely related to three H4N6 viruses from LPM, suggesting a potential AIV link between farms and LPMs. Additionally, the HA, NA, PB2, NP, and NS genes of two other H4N6 viruses isolated in LPM clustered with that of MH-2/H4N6. However, the remaining genes were more closely related to other sublineages, suggesting that MH-2/H4N6-originated viruses reassorted with other viruses in LPM. All H4 viruses replicated in mouse lungs without prior adaptation and all viruses replicated and transmitted among ducks. 29-1/H4N2, MH-2/H4N6, and 420-2/H4N6 viruses caused systemic infection in infected ducks. However, most of the viruses were not adapted in chickens. The present results indicate a potential correlation of AIV between LPMs and farms and suggest that active surveillance of AIV in LPM is warranted in China.

Avian influenza (AI), an infectious disease caused by influenza A viruses, is a threat to human and animal health. Wild birds and waterfowl are believed to be the natural hosts and reservoirs of avian influenza viruses (AIVs)^1^. AIVs are classified into highly-pathogenic AIVs (HPAIVs) and low-pathogenic AIVs (LPAIVs). H4 subtype AIVs, belonging to the LPAIVs, have been circulating in China and other countries^1^,^2^,^3^,^4^. H4 AIV has a wide host range, including chickens, turkeys, shorebirds, psittacine birds, seals, and pigs^5^,^6^,^7^. Animals infected with H4 usually remain asymptomatic while carrying the viruses^8^. However, one H4N6 isolate was reported to cause systemic infection and death in chickens^2^. Moreover, cross-species infections of H4 subtype have also occurred sporadically. An H4N6 subtype AIV was isolated from pigs with pneumonia in 1999^7^. Specific antibodies against H4 subtype AIV were detected in the sera of swine and in people working in chicken/turkey farms^9^,^10^,^11^. These data indicate that the H4 subtype virus may be capable of cross-species transmission. Thus, surveillance of H4 subtype AIV is warranted.

Live poultry markets (LPMs) are believed to be the source of AIV in China^12^. Commingling of multi-species birds in LPM provides an environment for virus reassortment and cross-species infection^13^,^14^,^15^. LPMs have been the source of many H7N9 human infections^14^,^16^. However, the ecology of influenza virus in LPM, the source of AIV in LPM, and the potential link between LPM and farms remain largely unclear^13^,^15^,^17^. In this study, we isolated an H4N6 subtype influenza virus from a diseased duck farm in Shanghai and then conducted active surveillance in the LPM to trace the H4 subtype AIVs. To understand the evolution of H4 AIV in duck farms and LPMs, the phylogenetic relationship and pathogenicity of all H4 isolates from ducks were evaluated in this study. The present results showed that multiple sublineages of H4 subtype influenza viruses co-circulated and reassorted with other influenza viruses in LPM. MH-2/H4N6 virus isolated from diseased duck farm had close relationship with certain H4N6 viruses isolated from LPM, suggesting a potential AIV link between farms and LPMs. Moreover, all isolated H4 viruses replicated in mice without prior adaptation, and certain strains gained direct-contact transmission in chickens, which suggested that duck-original H4 has adapted in chickens and posed potential threat to mammalian host.

## Materials and Methods

### Virus isolation

Respiratory disease was observed in a duck farm in Shanghai, China, in 2009. Subsequently, an H4N6 virus was isolated from oropharyngeal swab samples of the diseased ducks. Since LPM is the major place where live ducks are traded in Shanghai, active surveillance was conducted in the LPM in 2009 to investigate the epidemiology of the H4. Totally, 3,787 oropharyngeal swab samples were collected from ducks and chickens in LPM. Each sample was suspended in antibiotic solution in phosphate-buffered saline (PBS) containing 1,000 U/ml penicillin and 1,000 U/ml streptomycin and centrifuged at 13,800 g for 10 min. The filtered supernatants were inoculated into the allantoic cavity of 9-day-old specific pathogen-free (SPF) embryonated chicken eggs and incubated at 37 ˚C. Allantoic fluid from the incubated eggs was harvested 72 h after inoculation. An HA assay was conducted with 0.5% packed chicken red blood cells and HA-positive samples were subtyped by using an HI assay with anti-sera against AIVs (H1, H3, H4, H5, H6, H7, H9, H10 and H11) and RT-PCR using influenza-specific primers as described previously^18^,^19^. All isolated viruses were purified in 10-day-old SPF embryonated eggs by limiting dilution assay. The virus titers were determined in embryonated eggs and the 50% embryo infectious dose (EID_50_) was calculated by using the Reed and Muench method^20^.

### Genome sequencing and phylogenetic analysis

Viral RNA was extracted and converted to cDNA using reverse transcriptase M-MLV (TaKaRa, Dalian, China) in a final volume of 20 μl containing: 5 μl RNA, 4 μl 5 × RT buffer, 2 μl dNTPs (10 mM), 2 μl 10 μM specific primer (5′-AGCAAAAGCAGG-3′), 0.5 μl MLV reverse transcriptase, 0.5 μl RNase inhibitor (TaKaRa, Dalian, China), and 6 μl diethylpyrocarbonate (DEPC)-treated water. The reaction was incubated at 42 ˚C for 1 h followed by incubation at 70 ˚C for 15 min. The eight fragments of each virus were amplified using universal primers, as described previously^21^. Reaction mixtures were loaded onto agarose gels for electrophoresis after amplification, and the bands at expected fragment sizes were purified using a DNA Gel Extraction Kit (Axygen, Hangzhou, China) and sequenced by GENWIZ Biotechnology Company. Nucleotide sequences were assembled and analysis was conducted by using the programs in the DNASTAR package (DNASTAR Inc, USA). Phylogenetic trees were constructed using the neighbor joining method by MEGA6.0^22^ with 1,000 bootstrap replicates. The reference sequences used for genetic comparison were obtained from the GenBank database.

### Pathogenicity in ducks and chickens

To assess the pathogenicity of the isolated H4 AIVs in ducks and chickens, groups of 12-week-old influenza virus seronegative shelducks and 6-week-old SPF chickens were used. Six birds were inoculated intranasally with the isolated H4 viruses at 10^6^ EID_50_ in 0.1 ml PBS. To assess the transmissibility of H4 virus in ducks or chickens, three naïve ducks and chickens were co-mingled with infected birds. All birds were observed daily for clinical signs until the end of the experiment (14 days). Oropharyngeal and cloacal swabs were collected at 2, 4, and 6 days post infection/contact (dpi/dpc) from the inoculated and contact birds for detection of virus shedding. To elucidate virus replication and distribution in the infected birds, three birds from each group were euthanized at 3 dpi and tissue samples (trachea, lung, pancreas, spleen, kidney, and bursa of fabricus of the chicken) were collected for virus titration. The remaining birds were euthanized at the end of the experiment. Serum samples were collected at necropsy for seroconversion tests.

### Pathogenicity in mice

To investigate the pathogenicity of the H4 isolates in mice, groups of eleven 6-week-old BALB/c mice (Vital River Laboratories, Beijing, China) were inoculated intranasally with the viruses at 10^6^ EID_50_ in 50 μl PBS. Eleven other BALB/c mice were inoculated intranasally with 50 μl PBS as mock group. To evaluate viral replication in the respiratory tract and other organs, three mice from each group were euthanized on 3 and 5 dpi. At necropsy, the heart, liver, lung, nasal turbinate, spleen, kidney, and brain tissues were collected and titrated in 10-day-old embryonated chicken eggs. The remaining mice were observed for clinical signs and their body weight was measured until 14 dpi, the serum samples were collected from mice at 14 dpi. For histopathologic examination and immunohistochemistry (IHC), lung samples were fixed in 10% buffered formalin, processed, and stained with hematoxylin and eosin (H&E). An anti-influenza A antibody against the nucleoprotein (Genscript, USA) was used for IHC staining as described previously^23^.

### Ethical statement and statistical analyses

All animals were cared for in accordance to the guidelines of the Animal Care and Use Committee of Shanghai Veterinary Research Institute. Animal studies were approved by Chinese Academy of Agricultural Science (Permit number: SHVRI-Po-0120). Data were analyzed using analysis of variance (ANOVA) in GraphPad Prism version 6.0 (GraphPad software Inc, CA); a p-value < 0.05 was considered statistically significant.

## Results

### Virus isolation and identification

In 2009, a mild respiratory disease was observed in duck farms in Shanghai. Oropharyngeal swab samples were collected from the diseased ducks for virus isolation. An H4N6 subtype AIV was isolated and designated as A/duck/Shanghai/MH-2/2009 (H4N6, MH-2/H4N6). To trace the origin and distribution of this H4 subtype virus, an active surveillance program was conducted in LPM in Shanghai. A total of 188 AIV viruses were isolated from LPM of Shanghai in 2009, seven out of these isolates are H4 subtype AIV (six H4N6 and one H4N2) with isolation rate of 0.2%. All seven H4 AIVs were isolated from domestic ducks, which were provisionally designated as A/duck/Shanghai/29-1/2009 (H4N2, 29-1/H4N2), A/duck/Shanghai/44-2/2009 (H4N6, 44-2/H4N6), A/duck/Shanghai/46-2/2009 (H4N6, 46-2/H4N6), A/duck/Shanghai/67-2/2009 (H4N6, 67-2/H4N6), A/duck/Shanghai/408-1/2009 (H4N6, 408-1/H4N6), A/duck/Shanghai/420-2/2009 (H4N6, 420-2/H4N6), and A/duck/Shanghai/421-2/2009 (H4N6, 421-2/H4N6). Viruses were amplified in SPF chicken embryonated eggs. The genomes of all eight H4 isolates were sequenced and deposited in GenBank with accession numbers of KX162591 - KX162654.

### Molecular analyses

The eight H4 AIV isolates all shared the same amino acid sequence PEKASR/GLF at the HA cleavage site between HA1 and HA2, indicating that all viruses are low pathogenic influenza viruses. All H4 AIVs had 226Q and 228 G (according to H3 numbering) at the receptor binding region, implying that they retained typical avian virus-like receptor specificity (sialic acid -2,3-NeuAcGal). No AIV had a deletion in the NA stalk regions. No mammalian adaptation-associated mutations were observed in the PB2 gene (158E, 271 T, 590 G, 591Q, 627E, and 701D). F26, A/I27, T/V30, N31, E34, and F38 in the M2 protein are amantadine resistance markers. No such substitutions were found, except that four viruses (MH-2/H4N6, 44-2/H4N6, 46-2/H4N6, and 67-2/H4N6) had a V27I substitution. All of these H4 viruses had a full-length PB1-F2 protein of 90 amino acids except for 29-1/H4N2 (with 58 amino acids). An N66S mutation was found in PB1-F2 of 421-2/H4N6, which could increase the virulence of influenza A virus by inhibiting the early interferon response^24^,^25^.

### Phylogenetic analyses

**Figure 1:**
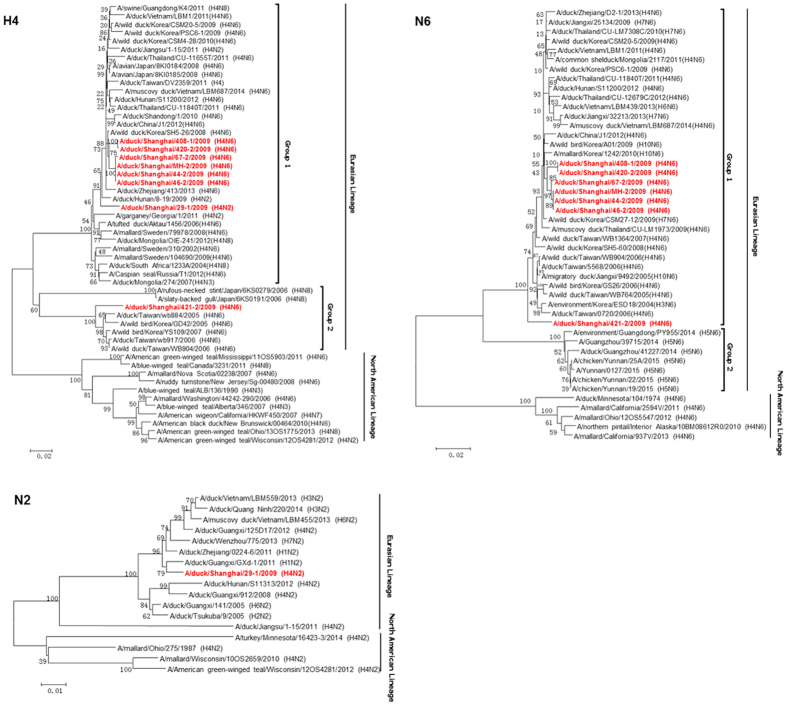
Phylogenetic trees of the H4, N6, and N2 genes of the eight H4 avian influenza viruses. Phylogenetic trees were generated by using the neighbor-joining method and bootstrapped with 1,000 replicates using MEGA6 software version 6.05. Phylogenetic trees were based on the comparison of nucleotide sequences of the H4 avian influenza viruses (AIVs) isolated in this study to reference AIV sequences published in GenBank. Isolates are highlighted in red. The scale bar represents the distance unit between sequence pairs.

The genetic diversity of the H4 AIVs was characterized based on a phylogenetic analysis of eight genes. Standard clade nomenclature system for H5 HA is used for H4 genes classification, which set boundaries of pairwise nucleotide distances >1.5% for between clades and <1.5% for within clades^26^. All the isolates clustered in the Eurasian lineage and were phylogenetically closely related to avian strains isolated from waterfowls. Phylogenetic analysis of the HA genes indicated that these strains were divided into two sublineages (group 1 and group 2), with an average nucleotide distance between the two groups of 13%. The MH-2/H4N6 HA was closely related to five H4N6 viruses isolated from LPM (group 1), while 421-2/H4N6 was located in group 2 in the HA phylogenetic tree (Fig. 1). All N6 genes were clustered in the Eurasian lineage, and were closely related to N6 genes circulating in Eastern China and Korea. Similarly, two sublineages were found in the phylogenetic tree of N6 gene (the nucleotide distance between groups of NA is 12%). The N6 gene of 421-2/H4N6 did not cluster with the other six H4 viruses (Fig. 1). N2 gene of 29-1/H4N2 was grouped into the Eurasian lineage and was closely related to H1N2 circulating in Eastern China (Fig. 1).

**Figure 2:**
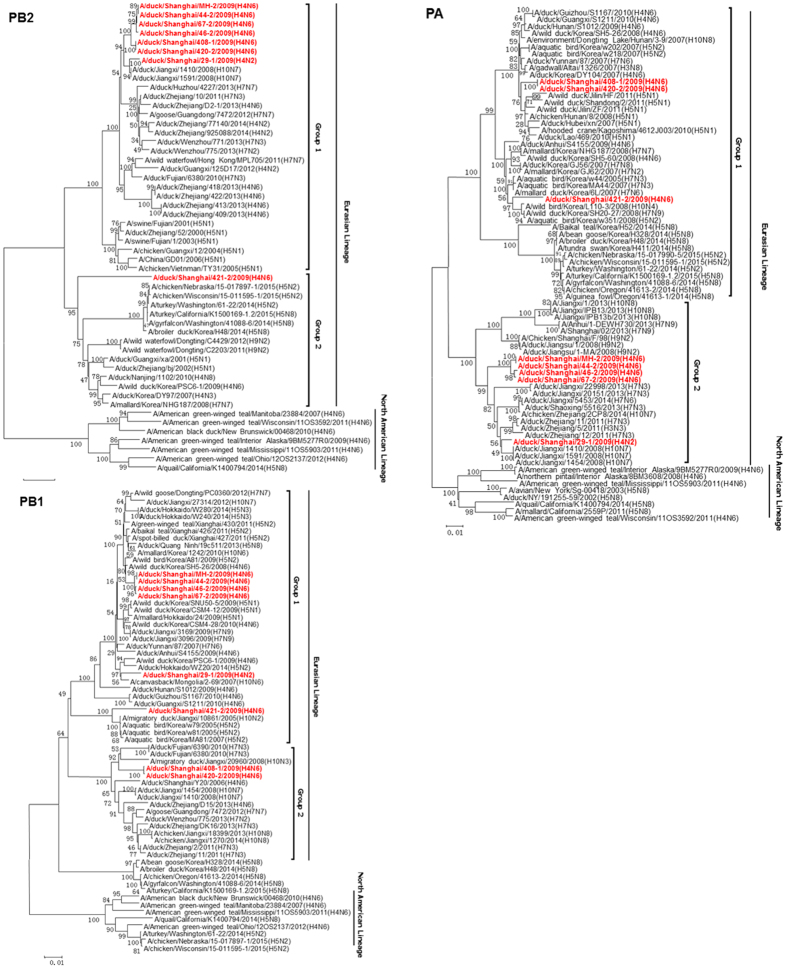
Phylogenetic trees of the PB2, PB1, and PA genes of the eight H4 influenza viruses. Phylogenetic trees were generated by using the neighbor-joining method and bootstrapped with 1,000 replicates using the MEGA6 software version 6.05. Phylogenetic trees were based on the comparison of nucleotide sequences of the H4 avian influenza viruses (AIVs) isolated in this study to reference AIV sequences published in GenBank. Isolates are highlighted in red. The scale bar represents the distance unit between sequence pairs.

**Figure 3:**
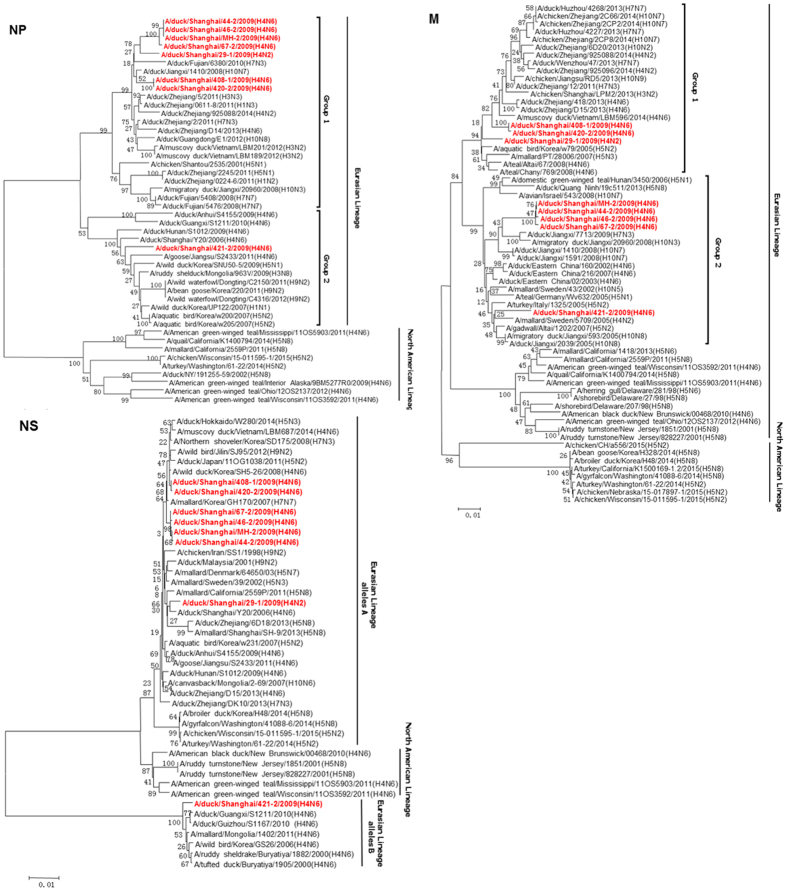
Phylogenetic trees of the NP, M, and NS genes of the eight H4 influenza viruses. The tree was generated by the neighbor-joining method and bootstrapped with 1,000 replicates using the MEGA6 software version 6.05. Phylogenetic trees were based on the comparison of nucleotide sequences of the H4 avian influenza viruses (AIVs) isolated in this study to reference AIV sequences published in GenBank. Isolates are highlighted in red. The scale bar represents the distance unit between sequence pairs.

Phylogenic analysis of internal genes showed that all eight H4 isolates belong to the Eurasian lineage (Figs 2 and 3). The PB2 and NP genes of MH-2/H4N6 were closely related to all H4 viruses isolated from LPM, except for 421-2/H4N6. Other internal genes (PB1, PA, M, and NS) of MH-2/H4N6 were clustered with those of 44-2/H4N6, 46-2/H4N6 and 47-2/H4N6 in the phylogenetic trees, suggesting that all the four viruses (44-2/H4N6, 46-2/H4N6, 47-2/H4N6 and MH-2/H4N6) shared the same progenitor. However, the PB1, PA, and M genes of 408-1/H4N6 and 420-2/H4N6 were located in different sublineages (Figs 2 and 3). The PB1 and M genes of 408-1/H4N6 and 420-2/H4N6 shared high identity with H10N7 influenza viruses. The PA gene grouped with H5N8, which has emerged in USA and Korea, indicating that reassortment occurred between H4N6 with other subtype influenza viruses in LPM. The 421-2/H4N6 HA, NA, PB2, PA and NP genes were genetically distinct from MH-2/H4N6. 421-2/H4N6 possessed allele B NS gene as described in a previous study^27^, while the other isolates possessed NS genes of allele A (Fig. 3). These data suggest that different H4 lineages co-circulated in LPM. The PB2 and PA genes of 421-2/H4N6 were closely related to the highly pathogenic H5N8 virus (Fig. 2). The PA genes of 408-1/H4N6 and 420-2/H4N6 grouped with those of highly pathogenic H5N1 viruses while the 29-1/H4N2 PA gene was closely related to the H10N7 virus. Other H4 isolates shared high homology with H7N3 virues (Fig. 2).

### Pathogenicity in ducks and chickens

To assess the pathogenicity of the isolated H4 AIVs in ducks and chickens, 12-week-old shelducks and 6-week-old chickens were infected intranasally with the H4 isolates at a dose of 10^6^ EID_50_. None of the H4 AIVs induced clinical signs of illness in ducks or chickens. All eight viruses replicated in ducks (Table 1). Three viruses (29-1/H4N2, MH-2/H4N6, and 420-2/H4N6) caused systemic infection in ducks, with virus detected in all organs including lung, trachea, spleen, kidney, and pancreas. However, no infected duck died. HI test showed that all infected ducks seroconverted at the end of the study.

In chickens, H4 viruses replicated to lower titers as compared with titers in ducks (Table 2). Two H4 viruses (67-2/H4N6, 408-1/H4N6) did not replicate in chickens, no virus was recovered from all tissue samples. Two viruses (44-2/H4N6, 420-2/H4N6) had broad tissue tropism in chickens but with low viral titers. 420-2/H4N6 infected and contact chickens seroconverted at the end of this study, seroconversion was observed in other viruses infected chickens, but not the contact chickens.

**Figure 4:**
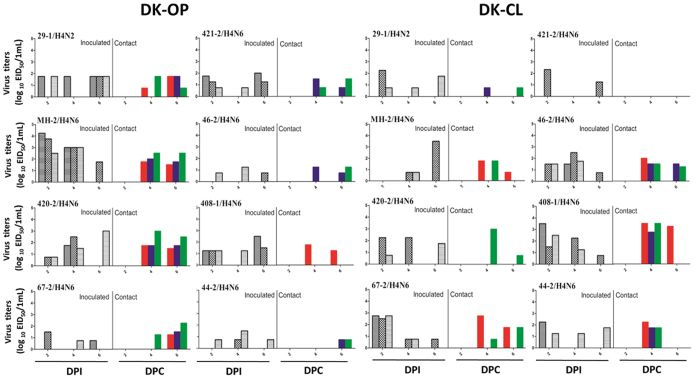
Virus titers in oropharyngeal and cloacal swabs of infected ducks. Six shelducks (12 week-old) were inoculated intranasally with the viruses at 10^6^ EID_50_. Three naïve ducks were introduced into the isolators later that day. Virus shedding in the respiratory and gastrointestinal tracts of both challenge and contact groups was monitored using oropharyngeal (OP) and cloacal (CL) swabs, respectively, taken at 2, 4, and 6 (4 and 6 dpc. for the contact group) days post-inoculation (p.i).

**Figure 5:**
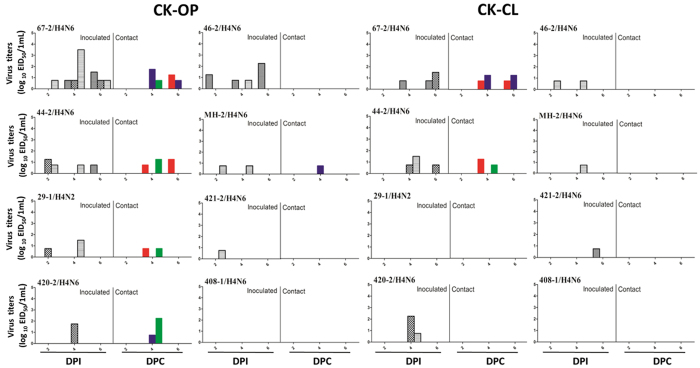
Virus titers in oropharyngeal and cloacal swabs of infected chickens. Six chickens (6 weeks-old) were inoculated intranasally with the viruses at 10^6^ EID_50_. Three naïve chickens were introduced into the isolators later that day. Virus shedding in the respiratory and gastrointestinal tracts of both challenge and contact groups was monitored using oropharyngeal (OP) and cloacal (CL) swabs, respectively, taken at 2, 4, and 6 (4 and 6 dpc. for the contact group) days post-inoculation (p.i.).

All infected ducks shed virus through oropharyngeal and cloacal routes to different extents, and all viruses transmitted among ducks (Fig. 4). All contact ducks seroconverted except for 421-2/H4N6. However, most infected chickens shed virus at lower titers (Fig. 5). Five out of eight AIVs were recovered in oropharyngeal swabs of contact chickens. Only two viruses were detected in cloacal swabs of contact chickens, which suggests that these H4 viruses have limited ability to replicate in chickens.

### Pathogenicity in mice

**Figure 6:**
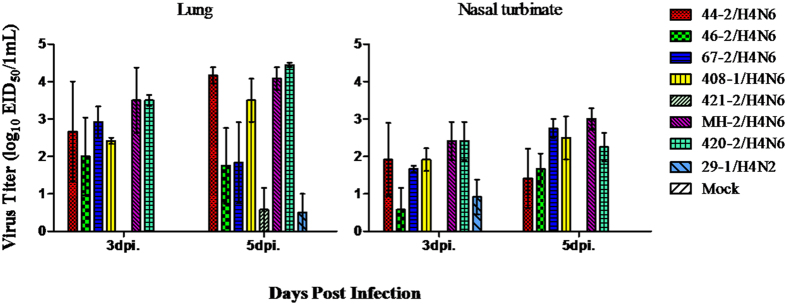
Replication of influenza virus in nasal turbinate and lung of inoculated mice. Mice were inoculated intranasally with the virus at 10^6^ EID50. The nasal turbinates and lungs were harvested at 3 and 5 dpi. The virus titers are shown as (log_10_EID50 per 0.1 ml) of the pooled sample.

**Figure 7:**
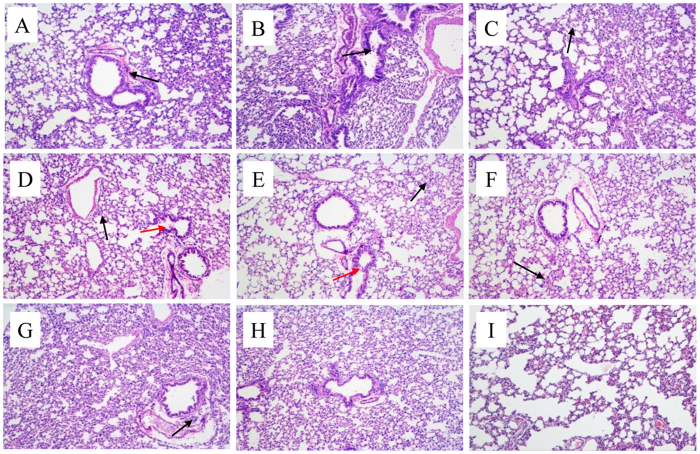
Microscopic lung sections from mice inoculated with H4 viruses at 5 dpi. (**A**–**D**): 29-1/H4N2, MH-2/H4N6, 44-2/H4N6, 46-2/H4N6; E-H): 67-2/H4N6, 408-1/H4N6, 420-2/H4N6, 421-2/H4N6; (**I**): Mock. Lungs were harvested at 5 dpi from mice inoculated intranasally with 10^6^ EID50 viruses. The black arrow represents infiltration of inflammatory cells, and the red arrow indicates mild detachment of bronchial epithelial cells.

**Figure 8:**
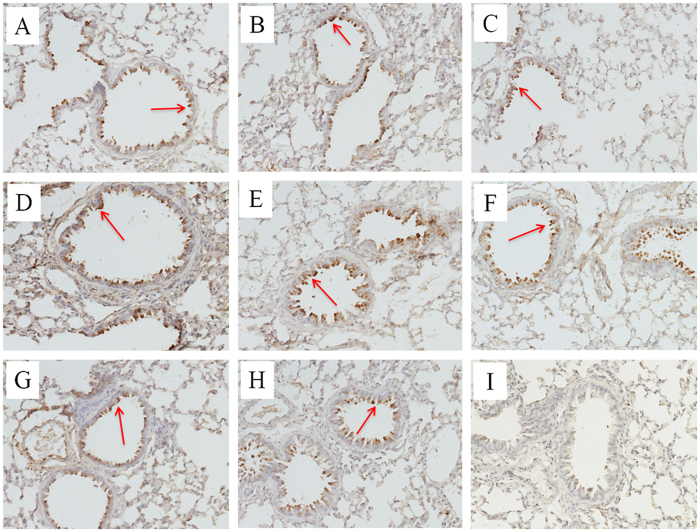
IHC staining of microscopic lung sections from mice infected with H4 viruses at 5 dpi. Positive immunohistochemical reactions were observed in lung sections of mice after infected with the H4 viruses, (**A**–**D**): 29-1/H4N2, MH-2/H4N6, 44-2/H4N6, 46-2/H4N6; E-H): 67-2/H4N6, 408-1/H4N6, 420-2/H4N6, 421-2/H4N6. No antigen was found in lung sections of mock mice, (**I**): Mock. Anti-influenza A antibody against nucleoprotein (Genscript, USA) was used for IHC staining.

No H4 virus induced clinical signs in infected mice and only slight weight loss was observed at 1 dpi. Six out of eight H4 isolates replicated in lungs and nasal turbinates without prior adaptation (Fig. 6), while no virus was detected in other tissue (heart, liver, brain, spleen, and kidney) of mice, indicating no virus caused systemic infection and all mice did not seroconvert at the end of the mice study. 421-2/H4N6 and 29-1/H4N2 replicated to significantly lower titers than the other viruses in both upper and lower respiratory tracts. Based on histopathological examination, all H4 viruses caused mild bronchointerstitial pneumonia, characterized by infiltration of the alveolar lumen with neutrophils and damage of the alveolar epithelium (Fig. 7). The NP antigen was observed through IHC staining of lung sections of mice after infection, suggesting that all H4 viruses replicated in mouse lungs (Fig. 8).

## Discussion

LPMs are considered to be a major source of AIV dissemination and interspecies transmission in China. Co-mingling of different birds (chickens, ducks, quails, guinea fowl, and pigeons etc.) in LPM enhances the reassortment potential of AIV. Close contact between humans and the LPM provides conditions for cross-species AIV transmission^28^,^29^. For instance, most of the H7N9 infected humans had prior exposure to LPM, which was identified as the source of human H7N9 infection cases^30^,^31^. In this study, an active surveillance was conducted in LPM and poultry farms. An H4N6 virus that caused disease in a duck farm was closely related to H4 viruses circulating in LPM.

Here, we have reported the isolation of a H4 isolate (MH-2/H4N6) from mallards in a farm and traced their genetic and pathogenic characteristics using genetic analyses and animal experiments. Seven other H4 AIVs were isolated from LPMs in Shanghai. The NA genes of all seven H4N6 isolates were located in group 1, though 421-2/H4N6 was distantly related to the other six H4 isolates. The HA genes of the other H4 isolates fell into group 1, which contains the H4 viruses recently isolated in Asia. However, the HA gene of 421-2/H4N6 belonged to group 2, which contains H4 strains isolated in Asia during 2005–2007. Similarly, the 421-2/H4N6 PB2, NP, and NS genes were only distantly related to the other six H4 isolates. Thus, two H4 lineages with different reassortant patterns are co-circulating in LPMs. The MH-2/H4N6 isolated from a diseased duck farm was closely related to five H4N6 viruses from LPM (Fig. 1), suggesting extensive reassortment in LPM and a potential link between farm and LPM. For instance, 420-2/H4N6 and 408-1/H4N6 possessed the PB1, PA, and M genes from viruses in group 2, while the remaining genes were more similar to that of the MH-2/H4N6 virus. Overall, extensive reassortment among different subtype influenza viruses occurred in LPM in Shanghai.

Aquatic birds are the natural reservoir of influenza virus. The virus replicates in bird intestines but does not cause disease^32^,^33^. Since 2005, highly pathogenic H5N1 virus started to infect and kill wild birds in China, causing concern about the pandemic potential of H5N1^34^. However, the low pathogenic influenza viruses do not usually cause disease in birds. Our pathogenicity studies of H4 in chickens and ducks showed that H4N6 replicated better in ducks than in chickens. Although the H4 viruses did not cause clinical signs of disease in ducks, 29-1/H4N2, MH-2/H4N6, and 420-2/H4N6 caused systemic infection in ducks in the experimental study. Virus was isolated from lung, trachea, spleen, kidney, and pancreas, though at low titers. MH-2/H4N6 was isolated from a diseased duck farm, but did not cause any clinical signs of disease in experimentally infected ducks, suggesting that co-infection of H4N6 with other pathogens caused disease in the diseased duck farm. All H4 viruses were transmissible in ducks, but replicated and transmitted less efficiently in chickens, suggesting that the H4 viruses have adapted to ducks rather than to chickens. 421-2/H4N6 showed limited replication in chickens and mice, as compared with other strains. The 421-2/H4N6 HA, NA, PB2, NP and NS genes were distinct from the genes of the other six H4 isolates based on phylogenetic analyses, which might contribute to the inefficient replication of 421-2/H4N6.

Influenza viruses evolve over time through mutation and reassortment^35^. In this study, reassortments of H4 subtypes with other virus in LPM were observed. None of the AIVs induced clinical signs of disease or lethality to mice, but seven of the AIVs were recovered from the lungs at 3 and 5 dpi., demonstrating the potential for waterfowl-origin H4 AIVs to infect mice without prior adaptation. During their replication in mammals, H4 AIVs may acquire new mutations and become more virulent or more transmissible^36^. H4 AIV infection has occurred in humans working with chickens^9^,^10^, indicating that H4 has the potential to cross species barriers to infect humans. The acquisition of efficient respiratory droplet transmission among humans is a prerequisite for the outbreak of an influenza pandemic. According to a recent report, H4 viruses could transmit to guinea pigs via direct contact and respiratory droplets^36^.

Although H4 AIVs are low pathogenic AIVs, it is possible that they will eventually gain the ability to transmit efficiently through the accumulation of mutations and reassortments. Therefore, it is important to investigate further the mechanisms of H4 AIVs mutation and reassortment, to prepare for potential pandemics.

## Additional Information

**How to cite this article**: Shi, Y. *et al.* Characterizations of H4 avian influenza viruses isolated from ducks in live poultry markets and farm in Shanghai. *Sci. Rep.* **6**, 37843; doi: 10.1038/srep37843 (2016).

**Publisher's note:** Springer Nature remains neutral with regard to jurisdictional claims in published maps and institutional affiliations.

## Figures and Tables

**Table 1:** Virus titers in tissues of infected ducks.

Virus	Virus Titers (log_10_ EID_50_/1 ml)				
	Lung	Trachea	Spleen	Kidney	Pancreas
29-1/H4N2	2/3(1.63 ± 0.18)^a^	1/3 (1.75)	2/3(1.38 ± 0.38)	2/3(1.63 ± 0.53)	3/3(1.83 ± 0.63)
MH-2/H4N6	3/3(1.58 ± 0.14)	3/3(1.92 ± 0.29)	3/3(1.33 ± 0.14)	3/3(1.58 ± 0.14)	3/3(2.00 ± 0.66)
44-2/H4N6	0/3 (N.A.)	2/3(1.25 ± 0.71)	1/3(0.75)	0/3 (N.A.)	3/3(1.35 ± 0.26)
46-2/H4N6	1/3(0.75)	2/3 (1.50 ± 0.35)	1/3(1.50)	3/3(1.33 ± 0.14)	1/3(1.25)
67-2/H4N6	1/3(1.25)	0/3 (N.A.)	0/3 (N.A.)	1/3(0.75)	3/3(1.33 ± 0.14)
408-1/H4N6	3/3(1.50 ± 0.00)	3/3(1.50 ± 0.00)	0/3 (N.A.)	1/3(1.50)	3/3(1.20 ± 0.26)
420-2/H4N6	3/3(1.67 ± 0.52)	3/3(1.33 ± 0.14)	3/3(1.58 ± 0.14)	3/3(1.75 ± 0.50)	3/3(1.83 ± 0.38)
421-2/H4N6	0/3 (N.A.)	0/3 (N.A.)	0/3 (N.A.)	0/3 (N.A.)	3/3(1.33 ± 0.14)

Six shelducks (12 weeks-old) were inoculated intranasally with the viruses at a dose of 10^6^ EID_50_. Three from each group were euthanized at 3 dpi and tissue samples (trachea, lung, pancreas, spleen, and kidney) were obtained for virus titration.

^a^2/3 infected ducks were positive for virus detection, and the titer values were shown as (mean ± SEM); N.A.: virus was not detected in any duck.

**Table 2:** Virus titers in tissues of infected chickens.

Virus	Virus Titers (log_10_ EID_50_/1 mL)					
	Lung	Trachea	Spleen	Kidney	Pancreas	Bursa of fabricius
29-1/H4N2	1/3 (1.25)	0/3 (N.A.)	2/3 (1.13 ± 0.38)^a^	2/3 (075 ± 0.00)	3/3(1.42 ± 0.14)	3/3(2.00 ± 0.66)
MH-2/H4N6	0/3 (N.A.)	1/3 (0.75)	0/3 (N.A.)	1/3 (1.50)	3/3(1.25 ± 0.00)	2/3 (1.38 ± 0.18)
44-2/H4N6	3/3(1.33 ± 0.14)	3/3(1.42 ± 0.29)	2/3 (1.13 ± 0.53)	1/3 (1.50)	3/3 (1.33 ± 0.52)	1/3 (1.50)
46-2/H4N6	1/3(0.75)	0/3 (N.A.)	0/3 (N.A.)	1/3 (1.25)	2/3 (1.38 ± 0.18)	1/3(0.75)
67-2/H4N6	0/3 (N.A.)	0/3 (N.A.)	0/3 (N.A.)	0/3 (N.A.)	0/3 (N.A.)	0/3 (N.A.)
408-1/H4N6	0/3 (N.A.)	0/3 (N.A.)	0/3 (N.A.)	0/3 (N.A.)	0/3 (N.A.)	0/3 (N.A.)
420-2/H4N6	3/3(1.08 ± 0.58)	3/3(1.58 ± 0.14)	3/3(1.50 ± 0.25)	3/3(1.33 ± 0.52)	3/3(2.17 ± 0.38)	3/3(1.75 ± 0.66)
421-2/H4N6	0/3 (N.A.)	0/3 (N.A.)	0/3 (N.A.)	0/3 (N.A.)	0/3 (N.A.)	1/3 (0.75)

Six chickens (6 weeks-old) were inoculated intranasally with the viruses at a dose of 10^6^ EID_50_. Three chickens from each group were euthanized at 3 dpi, and tissue samples (trachea, lung, pancreas, spleen, kidney and bursa of fabricus) were obtained for virus titration.

^a^2/3 infected chickens were positive for virus detection, and the titer values were shown as (mean ± SEM); N.A.: virus was not detected in any chicken.
